# Network analysis of emotion regulation and reactivity in adolescents: identifying central components and implications for anxiety and depression interventions

**DOI:** 10.3389/fpsyt.2023.1230807

**Published:** 2023-10-05

**Authors:** Qian-Nan Ruan, Chun-Mian Chen, Jiang-Shun Yang, Wen-Jing Yan, Zhen-Xing Huang

**Affiliations:** ^1^Department of Psychiatry, Wenzhou Seventh People’s Hospital, Wenzhou, Zhejiang Province, China; ^2^Department of Psychology, Wenzhou University, Wenzhou, China

**Keywords:** difficulties in emotion regulation (DER), emotion reactivity (ER), adolescents, network analysis, anxiety, depression

## Abstract

Difficulties in emotion regulation (DER) and emotion reactivity (ER) are important causes and consequences of psychiatric disorders such as depression and anxiety, and previous research suggests that there are many interactions between them. Understanding the structure of their relationship, and which components may play a key role, will help provide insight into emotion disorders in adolescents and provide guidance for clinical interventions. In this study, we collected data from 483 adolescents and used network analysis methods to explore the relationship between DER and ER, specifically looking for core nodes. The results showed that “limited access to emotion regulation strategies” was the most central node in the network. Furthermore, by adding nodes for depression and anxiety to this network, we found that anxiety had the strongest relationship with ER, while depression had a stronger relationship with DER. Thus, our findings suggest that for anxiety disorders, the strong association with ER highlights a potentially promising area for intervention development, whereas for depression, the association with DER points to the possibility of clarifying emotions and exploring coping strategies, acknowledging the complex interplay between depressive and anxious symptoms.

## 1. Introduction

In contemporary clinical practice, there is an increasingly recognized importance of emotions in comprehending psychopathology. Difficulties in Emotion Regulation (DER), characterized by an absence of awareness, unwillingness to accept emotions, and the employment of ineffective regulation strategies, play a critical role. Individuals with DER are predisposed to endure prolonged or more intense negative emotions, such as depression and anxiety, in comparison to their counterparts without such difficulties (1, 2). The Difficulties in Emotional Regulation Scale (DERS) serves as an instrumental tool for assessing these issues, encompassing areas like emotional arousal, awareness, understanding, acceptance, and emotional interference (3, 4).

Emotion Reactivity (ER), which encompasses emotional sensitivity, intensity, and persistence in response to stimuli, is another critical aspect (5, 6). While early self-report measures centered on intensity, the Emotional Reactivity Scale (ERS) assesses all three dimensions (7, 8). A single underlying factor has been demonstrated to best encapsulate these components through factor analysis (8, 9).

DER is thought to be closely related to ER (10–12). Basic emotion and appraisal theories treat ER and emotion regulation as very different constructs. One study provided four distinct but related perspectives on ER and emotion regulation, including basic emotion, appraisal, psychological, and social construct models, providing a theoretical basis for formal distinctions on the spectrum (13, 14). Another study concluded that the relationship between dispositional emotional orientations and actual emotions and regulative behaviours is not one-to-one, but that ER may increase the likelihood that certain features of emotions and behaviours will act as regulators (15, 16). In turn, specific components of DER may contribute significantly to ER, such as emotion regulation strategies, which have been shown to help reduce adverse reactions to stress (17–19). The use of attentional regulation strategies requires the allocation of attention and other resources to solving difficulties or problems, or diverting attention away from what is emotionally distressing (20, 21). These studies suggest that limited access to emotion regulation strategies plays an important role in ER.

The core of this investigation pertains to the exploration of DER and ER during the turbulent phase of adolescence. This period is distinguished by escalated stress and heightened susceptibility to the emergence of psychopathologies, such as anxiety and depression (22, 23). The typical trajectory of ER may encounter substantial inflection during adolescence, mirroring an increase in reactivity and amplified sensitivity to stimuli (24, 25). Concurrently, emotional control development and regulation may coincide with physical maturation (26), with the adolescent brain’s evolution possibly influencing emotion regulation methodologies (27, 28). This developmental stage, characterized by substantial structural and functional shifts, includes pronounced growth in the prefrontal cortex, an area vital for executive function and emotion regulation (29). Moreover, rapid synaptic pruning and myelination enhance connectivity between various brain regions, notably the amygdala, a crucial component in emotional processing (30, 31). These neural correlates elucidate the dynamic equilibrium between emotional reactivity and control, illuminating the complex interplay of neural networks during this pivotal developmental stage.

Finally, the study’s objective is to scrutinize the network connectivity of DER and ER throughout adolescence. Employing a bottom-up, data-driven network analysis, the investigation will explore these connections. This approach aids in pinpointing key components in emotional dysfunction, with nodes representing depression and anxiety added to the network to evaluate the association between emotional dysfunction and prevalent depressive or anxiety disorders. Such insights will furnish valuable perspectives for subsequent clinical interventions.

## 2. Methods

### 2.1. Participants

The study encompassed data from 483 adolescents, aged 12–17 years, recruited from a psychiatric hospital (216 adolescents) and two secondary schools (267 adolescents). Post manipulation check, 115 participants were excluded, culminating in 368 participants eligible for data analysis (32, 33). The subjects completed the DERS, ERS, and HADS under guided supervision. Ethical adherence was maintained throughout, conforming to national and institutional human experimentation standards and the Declaration of Helsinki (revised 2008), with approval by the IRB of the Seventh People’s Hospital of Wenzhou (EC-KY-2022048).

### 2.2. Measures

The Difficulties in Emotion Regulation Scale (DERS) is a 36-item self-report scale measuring six aspects of emotion regulation (34). The DERS scale consists of six factors. Cronbach’s alpha values for the scale and the six subscales ranged from 0.88 to 0.96, with test–retest reliability ranging from 0.52 to 0.77 (2). The measure meets the required psychometric properties and is therefore suitable for measuring the level of DER in Chinese adolescents (35, 36).

The Emotion Reactivity Scale (ERS) is used to measure the level of an individual’s emotional response characteristics to events (8). The scale is divided into 21 questions across 3 dimensions and is used to assess an individual’s emotional sensitivity, intensity and persistence. The ERS has demonstrated good convergent, discriminant, and criterion-related validity.

The Hospital Anxiety and Depression Scale (HADS) assesses anxiety and depression, two emotions that often co-exist (37, 38). It is widely used because it is straightforward, quick and easy to administer. The HADS consists of a total of 14 questions, seven on depressive symptoms (i.e., the HADS-D) and seven on anxiety symptoms (the HADS-A). The correlations between these two subscales ranged from 0.40 to 0.74 (mean 0.56). The Cronbach’s alpha for the HADS-A was 0.83 and the Cronbach’s alpha for the HADS-D ranged from 0.67 to 0.90 (mean 0.82).

### 2.3. Network analysis

The study employed a Gaussian graphical model (GGM) for network analysis utilizing the R package qgraph (version 1.9.2) (39). The GGM was regularized through a graphical lasso, optimizing interpretability by minimizing redundancy and creating a sparse network (40). Selection of the λ-regularization parameter was guided by the Extended Bayesian Information Criterion (EBIC), with specifications lambdaGam = 0.25 and alphaGam = 0.25. Centrality measures, indicative of overall connectivity, were computed for each node, including strength, closeness, betweenness, and expected influence (41). The network components were categorized into two clusters: ER with three components and DER with six components. Multidimensional scaling (MDS) facilitated network visualization, representing variable proximity as distance between points in a low-dimensional space (42, 43). The merit of this method lies in the interpretation of distances between nodes as Euclidean distances (42). Furthermore, the study assessed centrality index stability by modeling a network from a subset of the data via case-removal bootstrap (*n* = 1,000), considering the index unstable if the correlation value significantly declined as participants were withdrawn. The r-package *bootnet* served to evaluate the network’s resilience through bootstrapping (39).

## 3. Results

Descriptive statistics in this study can be found in Table 1. There were some differences between the groups. For Depression, Anxiety, ERS, and DERS, females had a higher intensity than did males. For depression, *t* = 1.40, *p* < 0.01. For anxiety, *t* = 2.12, *p* < 0.001. For ERS, *t* = 3.97, *p* < 0.001. For DERS, *t* = 5.12, *p* < 0.001. The clinical and non-clinical groups are also show the differences. For depression, *t* = 3.35, *p* < 0.001. For anxiety, *t* = 2.08, *p* < 0.05. For ERS, *t* = 1.53, *p* = 0.126. For DERS, *t* = 2.79, *p* < 0.01.

**Table 1:** Descriptive statistics for the measurements.

			Depression		Anxiety		ERS		DERS	
		*N*	*M*	SD	*M*	SD	*M*	SD	*M*	SD
Gender	Female	205	8.17	5.00	9.21	4.79	48.96	20.73	109.58	29.11
	Male	160	6.76	4.67	7.09	4.92	40.18	21.27	94.60	25.80
Source	Clinical	206	8.30	5.39	8.76	5.36	46.64	22.10	106.65	30.41
	School	159	6.58	4.00	7.66	4.31	43.13	20.33	98.37	25.57
Family structure	Regular	271	6.56	4.57	7.41	4.59	41.97	21.11	98.17	27.40
	Single parent	36	9.03	4.44	9.86	5.61	51.14	20.94	110.39	27.06
	Reconstituted	17	10.53	4.32	10.82	4.94	52.47	19.05	110.29	30.14
	Orphan	1	12.00		12.00		67.00		152.00	
Economic status	Rich	3	2.33	1.53	4.67	4.73	29.33	22.74	78.67	17.56
	Wealthy	86	6.05	4.56	6.62	4.40	38.58	21.81	94.51	26.95
	Normal	224	7.42	4.61	8.38	4.90	45.49	20.67	102.83	28.13
	Poor	11	7.91	5.24	7.45	4.18	45.27	21.18	98.64	26.18

### 3.1. The network for DER and ER

**Figure 1:**
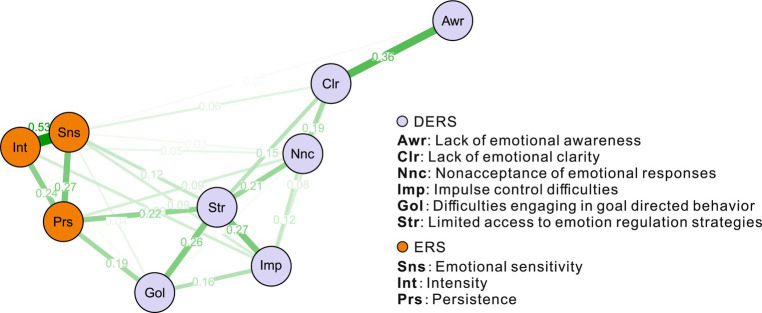
An estimated network structure based on 368 samples. The network shows the relationships among variables, including components from DERS and ERS. The edge weights are the regression coefficients, with regularization. The thickness of an edge reflects the magnitude of the relationship.

**Figure 2:**
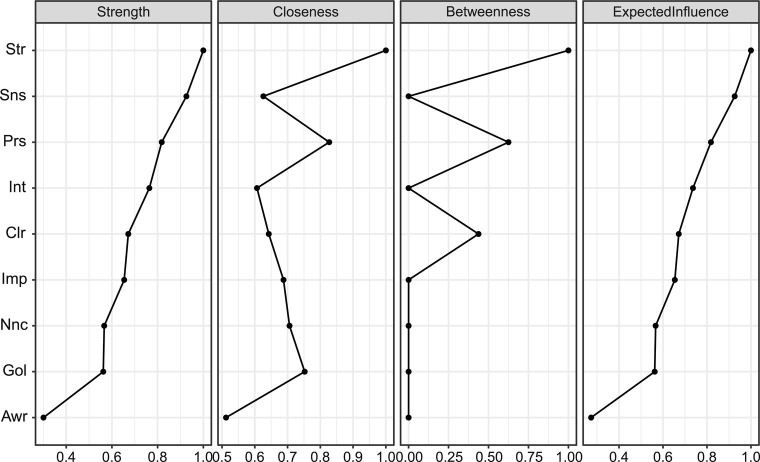
Centrality indices for the nodes of the male and female networks, including those for strength, betweenness, closeness, and expected influence.

In the network, approximately 9 of the 36 network edges (30.6%) were set to zero by regularization. Figure 1 illustrates that the ERS components are clustered on the left, relatively separate from the DERS components. The strong relationship between the ERS components reflects previous findings that a single underlying factor best explains the items (8, 9). The *Str* node is located at the centre of the network structure. It has a strong relative association with *Prs* (weight = 0.22) and *Sns* (weight = 0.12) in the ERS, suggesting that *Str* is the bridge between the ERS and DERS. In addition to *Awr* and *Str* nodes are also strongly associated with other nodes in the DERS component. The importance of *Str* in the network can also be seen in the centrality index below (see Figure 2).

The centrality index (Figure 2) shows the importance of *Str* in the network. Centrality indices, ordered by strength and normalized, ranged from 0 to 1. *Str* from the DER component was the most central, while *Sns* and Prs from the ERS component were second and third. However, a large index did not always mean the most connections. The *Sns* node had a high weight sum but few connections and was distant from other nodes. *Str* ranked first in all indices, indicating the highest weight sum, closest proximity to other nodes, and presence on the shortest path.

**Figure 3:**
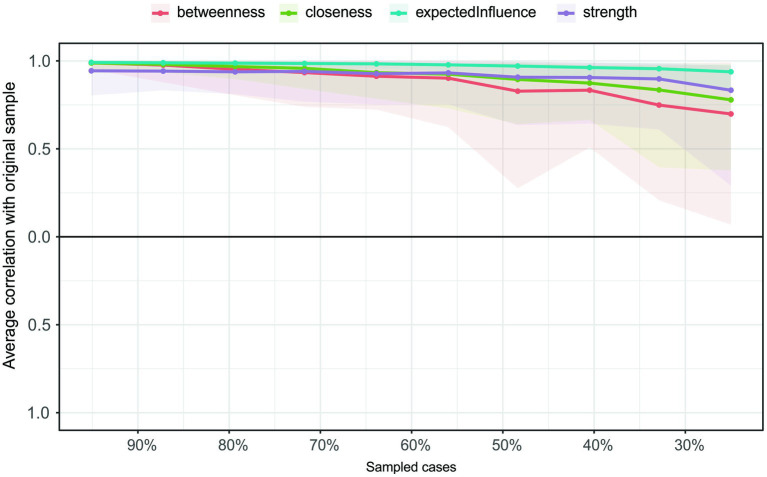
The average correlation coefficients between the remaining and original samples for the centrality indices of the network. Lines indicate the average correlation coefficients and areas show the coefficients ranging from the 2.5th quantile to the 97.5th quantile.

Stability was measured in terms of the maximum proportion of drops to maintain a correlation of 0.7 in at least 95% of the samples (see Figure 3). Correlations were calculated between the centrality indices of the networks from the selected and original samples. The value of the CS coefficient should preferably be above 0.5 and at least 0.25. In this research, the CS coefficient indicated that the betweenness (CS (cor = 0.7) = 0.44) and the closeness (CS (cor = 0.7) = 0.44), strength (CS (cor = 0.7) = 0.595) and expected influence (CS (cor = 0.7) = 0.75) were all stable in the subset cases. Therefore, we found that all indices were sufficiently stable.

### 3.2. The network of depression, anxiety, and emotion discrimination

Considering that emotional dysfunction are closely related to anxiety and depression, we added these two nodes to the network to further explore the clinical significance of the anxiety and depression crossover. In Figure 1 we observed that subscale nodes are strongly connected with each other, and previous studies showed that the three separate subscales in the ERS can also be explained by an underlying factor (8, 9). Therefore, to display a clear network, we included the total ERS score as a node.

**Figure 4:**
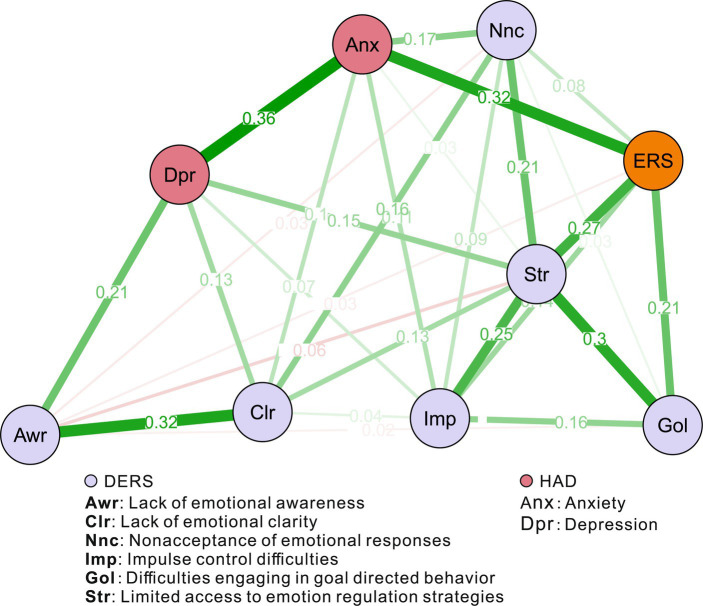
An estimated network structure of one ERS node, six DERS component nodes, one depression node, and one anxiety node, based on 368 samples. The edge weights are the regression coefficients, with regularization. The thickness of the edge reflects the magnitude of the relationship.

The estimated network structure can be seen in Figure 4, which shows that *Str* again remains in the centre, but is not directly connected to *Anx* and *Dpr*. It should be noted that the centrality indices in this network are much less meaningful because ERS, *Anx* and *Dpr* are the nodes representing the total scores of their scales, whereas *Str* is only one of the subscales of DERS. A subscale is naturally more correlated with the other subscales within the same scale, so the sum of the edge weights is high. The highest edge weight between *Anx* and the components of emotion dysregulation was for *Anx*-*ERS* at 0.32, a high weight as a regularized coefficient. Therefore, a promising intervention for anxiety symptoms may be the modulation of ER. In comparison, *Dpr* had no edge to ERS but connected to some DERS components, such as *Awr*, *Str*, and *Clr*. a promising intervention for depression symptoms may be the modulation of DER, such as in cases with a lack of emotion awareness and clarity and limited access to emotion regulation strategies.

## 4. Discussion

This study constructed a network of difficulties in Emotion regulation (DER) and emotion reactivity (ER) and found limited access to emotion regulation strategies to be central to the network structure. In addition, anxiety was closely associated with ER, and depression with DER components.

Emotion regulation strategies, such as rethinking situations, hiding emotions, or using humor, are associated with life satisfaction, positive emotions, depression, anxiety, and negative emotions (44, 45). These strategies involve increasing positive or decreasing negative emotions through changes in thoughts or behaviours (46). Limited access to emotion regulation strategies makes managing and controlling emotions difficult, and it is associated with the belief that there is nothing that can be done to effectively regulate emotions (2, 47). Improving access to strategies can help patients recover, as the ability to regulate emotions is a key intervention target for various mental health conditions (48). Cognitive behavioral treatments often rely on emotion regulation strategies for success (49). Emotion regulation problems, particularly lack of access to emotion regulation techniques, may predict suicidal ideation in various age groups (50–53). Clinicians should teach new emotion regulation strategies and encourage the use of personal strategies in different situations (54). As for emotion reactivity, similar to some previous studies (55), anxiety disorders are characterized by increased emotion reactivity. Further, there is a stronger association between anxiety and emotional reactivity than there is for depression (56).

This strong relationship between anxiety and depression may signify shared underlying psychological mechanisms, as supported by recent research on comorbidity between anxiety and depression (57). The association underscores the possibility of common regulatory deficits affecting both conditions and offers insights into transdiagnostic factors that could transcend traditional diagnostic categories (58). However, the pronounced linkage between anxiety and depression, in contrast to their connections with ERS and DERS components, highlights the complexity of these relationships, requiring further nuanced exploration. Care must be taken to distinguish between shared and unique components of emotion regulation relating to the disorders, a theme echoed in recent studies (59). Ultimately, the model invites a more refined understanding of adolescent emotional functioning, but this interpretation should be approached with caution.

The present study is not without limitations. A central concern stems from the cross-sectional design, which not only limits the ability to establish causal relationships between emotion regulation, anxiety, and depression but also hampers the potential to observe temporal changes and dynamic interactions over time in the studied variables. Additionally, the lack of clinical diagnoses and evaluation of specific interventions must be considered when interpreting the findings. The study may have overlooked certain critical variables or confounders that could influence emotion regulation, such as developmental factors, and co-occurring mental health conditions, which remain unassessed in the analysis. The complexity of the association between depressive and anxious symptoms also adds a layer of intricacy in understanding these relationships. Lastly, the interpretations drawn regarding the specific clinical interventions should be viewed with caution, as they could constitute interpretative leaps without more robust empirical support from longitudinal or experimental designs. Readers are encouraged to consider these findings as suggestive of potential avenues for intervention rather than definitive guides to treatment.

In summary, the present study explored the detailed relationships between DERS and ERS. The findings showed that “limited access to emotion regulation strategies” was the most central node in the DERS-ERS network. In addition, anxiety symptoms in adolescents are more strongly associated with emotion reactivity, highlighting a promising psychological process that may be explored for the development of interventions. Similarly, depressive symptoms are more associated with difficulties in emotion regulation, suggesting that facilitating access to emotional regulation strategies could be a beneficial path to explore in interventions. However, we acknowledge the high association between depressive and anxious symptoms and recognize that interventions must be tailored to the unique needs of each adolescent or case.

## Data availability statement

The raw data supporting the conclusions of this article will be made available by the authors, without undue reservation.

## Ethics statement

The studies involving humans were approved by IRB of the Seventh People’s Hospital of Wenzhou. The studies were conducted in accordance with the local legislation and institutional requirements. Written informed consent for participation in this study was provided by the participants’ legal guardians/next of kin.

## Author contributions

Q-NR and W-JY conceived and designed the experiments. Z-XH and J-SY performed the experiments and contributed to materials and analysis. W-JY analyzed the data. Q-NR and C-MC wrote the paper. All authors contributed to the article and approved the submittedversion.

## References

[1] Cengiz GF Gürel G. Difficulties in emotion regulation and quality of life in patients with acne. Qual Life Res. (2020) 29:431–8. doi: 10.1007/s11136-019-02318-2, PMID: 31605308

[2] Gratz KL Roemer L. Multidimensional assessment of emotion regulation and dysregulation: development, factor structure, and initial validation of the difficulties in emotion regulation scale. J Psychopathol Behav Assess. (2004) 26:41–54. doi: 10.1023/B:JOBA.0000007455.08539.94

[3] Hatkevich C Penner F Sharp C. Difficulties in emotion regulation and suicide ideation and attempt in adolescent inpatients. Psychiatry Res. (2019) 271:230–8. doi: 10.1016/j.psychres.2018.11.038, PMID: 30502560

[4] Yiğit İ Guzey YM. Psychometric properties of Turkish version of difficulties in emotion regulation scale-brief form (DERS-16). Curr Psychol. (2019) 38:1503–11. doi: 10.1007/s12144-017-9712-7

[5] Liu S You J Ying J Li X Shi Q. Emotion reactivity, nonsuicidal self-injury, and regulatory emotional self-efficacy: a moderated mediation model of suicide ideation. J Affect Disord. (2020) 266:82–9. doi: 10.1016/j.jad.2020.01.083, PMID: 32056950

[6] Wu H Gao Q Chen D Zhou X You J. Emotion reactivity and suicide ideation among Chinese adolescents: a longitudinal serial mediation model. Arch Suicide Res. (2023) 27:367–79. doi: 10.1080/13811118.2021.2000541, PMID: 34753413

[7] Ifeagwazi CM Egberi HE Chukwuorji JC. Emotional reactivity and blood pressure elevations: anxiety as a mediator. Psychol Health Med. (2018) 23:585–92. doi: 10.1080/13548506.2017.1400670, PMID: 29105504

[8] Nock MK Wedig MM Holmberg EB Hooley JM. The emotion reactivity scale: development, evaluation, and relation to self-injurious thoughts and behaviors. Behav Ther. (2008) 39:107–16. doi: 10.1016/j.beth.2007.05.005, PMID: 18502244

[9] Lannoy S Heeren A Rochat L Rossignol M Van der Linden M Billieux J. Is there an all-embracing construct of emotion reactivity? Adaptation and validation of the emotion reactivity scale among a French-speaking community sample. Compr Psychiatry. (2014) 55:1960–7. doi: 10.1016/j.comppsych.2014.07.023, PMID: 25176623

[10] Carthy T Horesh N Apter A Edge MD Gross JJ. Emotional reactivity and cognitive regulation in anxious children. Behav Res Ther. (2010) 48:384–93. doi: 10.1016/j.brat.2009.12.013 20089246

[11] McLaughlin KA Green JG Gruber MJ Sampson NA Zaslavsky AM Kessler RC. Childhood adversities and adult psychiatric disorders in the National Comorbidity Survey Replication II: associations with persistence of DSM-IV disorders. Arch Gen Psychiatry. (2010) 67:124–32. doi: 10.1001/archgenpsychiatry.2009.187, PMID: 20124112 PMC2847359

[12] Weissman DG Bitran D Miller AB Schaefer JD Sheridan MA McLaughlin KA. Difficulties with emotion regulation as a transdiagnostic mechanism linking child maltreatment with the emergence of psychopathology. Dev Psychopathol. (2019) 31:899–915. doi: 10.1017/S0954579419000348, PMID: 30957738 PMC6620140

[13] Gross JJ Feldman BL. Emotion generation and emotion regulation: one or two depends on your point of view. Emot Rev. (2011) 3:8–16. doi: 10.1177/1754073910380974, PMID: 21479078 PMC3072688

[14] Yih J Uusberg A Taxer JL Gross JJ. Better together: a unified perspective on appraisal and emotion regulation. Cognit Emot. (2019) 33:41–7. doi: 10.1080/02699931.2018.1504749, PMID: 30058449

[15] Davidson RJ Lewis DA Alloy LB Amaral DG Bush G Cohen JD. Neural and behavioral substrates of mood and mood regulation. Biol Psychiatry. (2002) 52:478–502. doi: 10.1016/S0006-3223(02)01458-0 12361665

[16] Hare TA Tottenham N Galvan A Voss HU Glover GH Casey BJ. Biological substrates of emotional reactivity and regulation in adolescence during an emotional go-nogo task. Biol Psychiatry. (2008) 63:927–34. doi: 10.1016/j.biopsych.2008.03.015, PMID: 18452757 PMC2664095

[17] Griffin SM Howard S. Individual differences in emotion regulation and cardiovascular responding to stress. Emotion. (2022) 22:331–45. doi: 10.1037/emo0001037, PMID: 34807696

[18] Gullo S Gelo OCG Bassi G Lo Coco G Lagetto G Esposito G. The role of emotion regulation and intolerance to uncertainty on the relationship between fear of COVID-19 and distress. Curr Psychol. (2023) 42:19658–69. doi: 10.1007/s12144-022-03071-5, PMID: 35496361 PMC9037968

[19] Moriya J Takahashi Y. Depression and interpersonal stress: the mediating role of emotion regulation. Motiv Emot. (2013) 37:600–8. doi: 10.1007/s11031-012-9323-4

[20] Nicolini D Korica M. Attentional engagement as practice: a study of the attentional infrastructure of healthcare chief executive officers. Organ Sci. (2021) 32:1273–99. doi: 10.1287/orsc.2020.1427

[21] Rothbart SAA Mary K. Temperament, development, and the big five In: The developing structure of temperament and personality from infancy to adulthood. ed. Rothbart MK. New York: Psychology Press (1995).

[22] Blake MJ Trinder JA Allen NB. Mechanisms underlying the association between insomnia, anxiety, and depression in adolescence: implications for behavioral sleep interventions. Clin Psychol Rev. (2018) 63:25–40. doi: 10.1016/j.cpr.2018.05.006, PMID: 29879564

[23] Wickersham A Barack T Cross L Downs J. Computerized cognitive behavioral therapy for treatment of depression and anxiety in adolescents: systematic review and meta-analysis. J Med Internet Res. (2022) 24:e29842. doi: 10.2196/29842, PMID: 35404263 PMC9039813

[24] Nelson BD Perlman G Hajcak G Klein DN Kotov R. Familial risk for distress and fear disorders and emotional reactivity in adolescence: an event-related potential investigation. Psychol Med. (2015) 45:2545–56. doi: 10.1017/S0033291715000471, PMID: 25851615 PMC4702485

[25] Liu S Oshri A Kogan SM Wickrama KAS Sweet L. Amygdalar activation as a neurobiological marker of differential sensitivity in the effects of family rearing experiences on socioemotional adjustment in youths. Biol Psychiatry Cogn Neurosci Neuroimaging. (2021) 6:1052–62. doi: 10.1016/j.bpsc.2021.04.017 33964518 PMC8568728

[26] Belcher BR Zink J Azad A Campbell CE Chakravartti SP Herting MM. The roles of physical activity, exercise, and fitness in promoting resilience during adolescence: effects on mental well-being and brain development. Biol Psychiatry Cogn Neurosci Neuroimaging. (2021) 6:225–37. doi: 10.1016/j.bpsc.2020.08.005, PMID: 33067166 PMC7878276

[27] Casey BJ Heller AS Gee DG Cohen AO. Development of the emotional brain. Neurosci Lett. (2019) 693:29–34. doi: 10.1016/j.neulet.2017.11.055, PMID: 29197573 PMC5984129

[28] Pozzi E Vijayakumar N Rakesh D Whittle S. Neural correlates of emotion regulation in adolescents and emerging adults: a meta-analytic study. Biol Psychiatry. (2021) 89:194–204. doi: 10.1016/j.biopsych.2020.08.006, PMID: 33268030

[29] Wellman CL Bollinger JL Moench KM. Chapter six-effects of stress on the structure and function of the medial prefrontal cortex: insights from animal models In: Clow A Smyth N, editors. International review of neurobiology, vol. 150. Stress and Brain Health: Across the Life Course: Academic Press (2020). 129–53. Available at: https://www.booksamillion.com/p/Stress-Brain-Health/Angela-Clow/9780128167526?id=8378507835726. 10.1016/bs.irn.2019.11.007 PMC9483990 32204829

[30] Bigot M Alonso M Houenou J Sarrazin S Dargél AA Lledo PM. An emotional-response model of bipolar disorders integrating recent findings on amygdala circuits. Neurosci Biobehav Rev. (2020) 118:358–66. doi: 10.1016/j.neubiorev.2020.07.037, PMID: 32739421

[31] Gothard KM. Multidimensional processing in the amygdala. Nat Rev Neurosci. (2020) 21:565–75. doi: 10.1038/s41583-020-0350-y, PMID: 32839565 PMC7714370

[32] Chmielewski M Kucker SC. An MTurk crisis? Shifts in data quality and the impact on study results. Soc Psychol Personal Sci. (2020) 11:464–73. doi: 10.1177/1948550619875149

[33] Oppenheimer DM Meyvis T Davidenko N. Instructional manipulation checks: detecting satisficing to increase statistical power. J Exp Soc Psychol. (2009) 45:867–72. doi: 10.1016/j.jesp.2009.03.009

[34] Victor SE Klonsky ED. Validation of a brief version of the difficulties in emotion regulation scale (DERS-18) in five samples. J Psychopathol Behav Assess. (2016) 38:582–9. doi: 10.1007/s10862-016-9547-9 PMC4882111 27239096

[35] Ling D Nan Z Xiaobin D. Reliability and validity of difficulties in emotion regulation normal scale in Chinese adolescent. Chin J Health Psychol. (2014) 22:363–6. doi: 10.13342/j.cnki.cjhp.201403021

[36] Ma Y Fang S. Adolescents’ mindfulness and psychological distress: the mediating role of emotion regulation. Front Psychol. (2019) 10:1358. doi: 10.3389/fpsyg.2019.01358, PMID: 31231292 PMC6567674

[37] Bjelland I Dahl AA Haug TT Neckelmann D. The validity of the hospital anxiety and depression scale: an updated literature review. J Psychosom Res. (2002) 52:69–77. doi: 10.1016/S0022-3999(01)00296-3 11832252

[38] Tomasoni D Bai F Castoldi R Barbanotti D Falcinella C Mulè G. Anxiety and depression symptoms after virological clearance of COVID-19: a cross-sectional study in Milan, Italy. J Med Virol. (2021) 93:1175–9. doi: 10.1002/jmv.26459, PMID: 32841387 PMC7461061

[39] Epskamp S Borsboom D Fried EI. Estimating psychological networks and their accuracy: a tutorial paper. Behav Res Methods. (2018) 50:195–212. doi: 10.3758/s13428-017-0862-1, PMID: 28342071 PMC5809547

[40] Foygel R Drton M. Extended Bayesian information criteria for Gaussian graphical models In: Advances in neural information processing systems, vol. 23: Curran Associates, Inc. (2010) (https://proceedings.neurips.cc/paper/2010/hash/072b030ba126b2f4b2374f342be9ed44-Abstract.html

[41] Borsboom D. A network theory of mental disorders. World Psychiatry. (2017) 16:5–13. doi: 10.1002/wps.20375, PMID: 28127906 PMC5269502

[42] Jones PJ Mair P McNally RJ. Visualizing psychological networks: a tutorial in R. Front Psychol. (2018) 9:1742. doi: 10.3389/fpsyg.2018.01742, PMID: 30283387 PMC6156459

[43] Mair J Wolf M Seelos C. Scaffolding: a process of transforming patterns of inequality in small-scale societies. Acad Manage J. (2016) 59:2021–44. doi: 10.5465/amj.2015.0725

[44] Hu T Zhang D Wang J Mistry R Ran G Wang X. Relation between emotion regulation and mental health: a meta-analysis review. Psychol Rep. (2014) 114:341–62. doi: 10.2466/03.20.PR0.114k22w4 24897894

[45] Menefee DS Ledoux T Johnston CA. The importance of emotional regulation in mental health. Am J Lifestyle Med. (2022) 16:28–31. doi: 10.1177/15598276211049771, PMID: 35185423 PMC8848120

[46] Hofmann SG. Interpersonal emotion regulation model of mood and anxiety disorders. Cogn Ther Res. (2014) 38:483–92. doi: 10.1007/s10608-014-9620-1, PMID: 25267867 PMC4175723

[47] Pepe M di Nicola M Moccia L Franza R Chieffo D Addolorato G. Limited access to emotion regulation strategies mediates the association between positive urgency and sustained binge drinking in patients with alcohol use disorder. Int J Ment Health Addict. (2022):1–14. doi: 10.1007/s11469-022-00807-z

[48] Moore R Gillanders D Stuart S. The emotional resources group: a mixed methods practice-based study of a transdiagnostic emotion regulation group intervention. Psychiatry Int. (2022) 3:297–312. doi: 10.3390/psychiatryint3040024

[49] Renna ME Quintero JM Fresco DM Mennin DS. Emotion regulation therapy: a mechanism-targeted treatment for disorders of distress. Front Psychol. (2017) 8:98. doi: 10.3389/fpsyg.2017.00098 28220089 PMC5292405

[50] Ciarrochi J Deane FP Anderson S. Emotional intelligence moderates the relationship between stress and mental health. Personal Individ Differ. (2002) 32:197–209. doi: 10.1016/S0191-8869(01)00012-5

[51] Rajappa K Gallagher M Miranda R. Emotion dysregulation and vulnerability to suicidal ideation and attempts. Cogn Ther Res. (2012) 36:833–9. doi: 10.1007/s10608-011-9419-2

[52] Tamás Z Kovacs M Gentzler AL Tepper P Gádoros J Kiss E. The relations of temperament and emotion self-regulation with suicidal behaviors in a clinical sample of depressed children in Hungary. J Abnorm Child Psychol. (2007) 35:640–52. doi: 10.1007/s10802-007-9119-2, PMID: 17530394

[53] Weinberg A Klonsky ED. Measurement of emotion dysregulation in adolescents. Psychol Assess. (2009) 21:616–21. doi: 10.1037/a0016669 19947794

[54] Neacsiu AD Smith M Fang CM. Challenging assumptions from emotion dysregulation psychological treatments. J Affect Disord. (2017) 219:72–9. doi: 10.1016/j.jad.2017.05.016, PMID: 28527311

[55] Kring AM Sloan DM. Emotion regulation and psychopathology: a transdiagnostic approach to etiology and treatment. New York: Guilford Press (2009).

[56] Watson D Naragon-Gainey K. Personality, emotions, and the emotional disorders. Clin Psychol Sci. (2014) 2:422–42. doi: 10.1177/2167702614536162, PMID: 25815243 PMC4370336

[57] Garabiles MR Lao CK Xiong Y Hall BJ. Exploring comorbidity between anxiety and depression among migrant Filipino domestic workers: a network approach. J Affect Disord. (2019) 250:85–93. doi: 10.1016/j.jad.2019.02.062, PMID: 30836284

[58] Young KS Sandman CF Craske MG. Positive and negative emotion regulation in adolescence: links to anxiety and depression. Brain Sci. (2019) 9:76. doi: 10.3390/brainsci9040076, PMID: 30934877 PMC6523365

[59] Chen S Bonanno GA. Components of emotion regulation flexibility: linking latent profiles to depressive and anxious symptoms. Clin Psychol Sci. (2021) 9:236–51. doi: 10.1177/2167702620956972

